# Pulmonary Embolism in Patients with End-Stage Kidney Disease Starting Dialysis

**DOI:** 10.1001/jamanetworkopen.2025.0848

**Published:** 2025-03-17

**Authors:** Kunal N. Patel, Wan-Chi Chan, Vivek Bhat, Monil M. Majmundar, Harsh Mehta, Cyrus Munguti, Kartik Munshi, Sri G. Yarlagadda, Gaurav M. Parmar, Aditya M. Sharma, Daniella Kadian-Dodov, Lewis G. Satterwhite, Jinxiang Hu, Jordan Baker, MS, Kamal Gupta

**Affiliations:** 1 Department of Cardiovascular Medicine, University of Kansas Medical Center, Kansas City; 2 Department of Internal Medicine, SUNY Upstate Medical University, Syracuse, New York; 3 Division of Nephrology, University of Kansas Medical Center, Kansas City; 4 Department of Vascular Medicine, Massachusetts General Hospital, Harvard Medical School, Boston; 5 Department of Internal Medicine, University of Virginia, Charlottesville; 6 Zena and Michael A. Wiener Cardiovascular Institute, Mount Sinai Fuster Heart Hospital, Icahn School of Medicine at Mount Sinai, New York, New York; 7 Department of Pulmonary, Critical Care and Sleep Medicine, University of Kansas Medical Center, Kansas City; 8 Department of Biostatistics and Data Science, University of Kansas Medical Center, Kansas City

## Abstract

This cohort study examines the incidence and outcomes of pulmonary embolism as well as anticoagulant use among patients with end-stage kidney disease initiating dialysis.

## Introduction

End-stage kidney disease (ESKD) confers a high risk of pulmonary embolism (PE) and is associated with relatively poor outcomes.^1,2^ Furthermore, ESKD is associated with a greater risk of bleeding, complicating management with anticoagulation (AC).^1^ Despite the complexity, there is a paucity of data regarding PE in patients with ESKD. We retrospectively analyzed data from the US Renal Data System (USRDS) to study the incidence, AC use, and outcomes of PE in patients with ESKD initiating dialysis.

## Methods

Using USRDS data, we identified incident patients with ESKD initiating dialysis from 2011 to 2019. Included patients had fee-for-service Medicare coverage within the first 90 days of dialysis initiation (eFigure in Supplement 1). Patients with prior atrial fibrillation, deep vein thrombosis or PE, or AC use were excluded. Outcomes included 1-year PE hospitalization incidence, mortality (in-hospital and 30-day), AC use, inferior vena cava filter use, PE recurrence, and differences in outcomes between hemodialysis (HD) and peritoneal dialysis (PD) (eMethods, eTable in Supplement 1). Data extraction and analyses were performed from March to May 2024 using SAS, version 9.4 (SAS Institute Inc). Continuous variables were compared using a 2-sample *t*-test, and categorical variables were compared using Pearson χ^2^ test. Two-sided *P* < .05 was statistically significant. This cohort study was approved by the University of Kansas Medical Center's Institutional Review Board and USRDS with a waiver of informed consent because data were deidentified. We followed the STROBE reporting guideline.

## Results

We identified 288 073 patients with ESKD (mean [SD] age, 68.7 [12.4] years; 161 752 males [56.2%]) who were initiating dialysis (Table). The 1-year PE incidence was 0.84% or 942 per 100 000 person-years, increasing from 925 to 1016 per 100 000 person-years between 2011 and 2019 (*P* = .05). The 3-year incidence was 1.7%. In-hospital and postdischarge 30-day mortality rates were 13.7% and 13.1%, respectively, with no significant temporal trends.

**Table.** Baseline Characteristics of Patients With ESKD and Those With PE

Characteristic	Patients with ESKD, No. (%)	
	All (N = 288 073)	With PE (n = 2377)
Sex		
Male	161 752 (56.2)	1146 (48.2)
Female	126 321 (43.8)	1231 (51.8)
Race^a^		
Asian and other^b^	17 410 (6.0)	72 (3.0)
Black	71 471 (24.8)	806 (33.9)
White	199 192 (69.2)	1499 (63.1)
Age, mean (SD), y	68.7 (12.4)	66.7 (14.1)
Age group, y		
<50	22 846 (7.9)	302 (12.7)
50-64	58 352 (20.3)	506 (21.3)
65-79	153 275 (53.2)	1170 (49.2)
≥80	53 600 (18.6)	399 (16.8)
ESKD cause		
Diabetes	144 406 (50.1)	1066 (44.9)
Hypertension	92 735 (32.2)	793 (33.4)
Other causes		
Total	50 932 (17.7)	518 (21.8)
Glomerulonephritis	16 753 (5.8)	165 (6.9)
Cystic kidney	4914 (1.7)	35 (1.5)
Urologic causes	4469 (1.6)	45 (1.9)
Not specified	24 796 (8.6)	273 (11.5)
Dialysis modality		
Hemodialysis	254 335 (88.3)	2181 (91.8)
Peritoneal dialysis	33 738 (11.7)	196 (8.3)
Comorbid conditions		
CAD	76 443 (26.5)	672 (28.3)
Cancer	27 419 (9.5)	304 (12.8)
CHF	106 745 (37.1)	988 (41.6)
COPD	37 055 (12.9)	426 (17.9)
CVA or TIA	32 320 (11.2)	270 (11.4)
Diabetes	184 476 (64.0)	1472 (61.9)
Hypertension	262 274 (91.0)	2154 (90.6)
PAD	47 291 (16.4)	425 (17.9)
Tobacco use	41 994 (14.6)	494 (20.8)

Abbreviations: CAD, coronary artery disease; CHF, congestive heart failure; COPD, chronic obstructive pulmonary disease; CVA, cerebrovascular accident; ESKD, end-stage kidney disease; PAD, peripheral arterial disease; PE, pulmonary embolism; TIA, transient ischemic attack.

^a^ Data on race reflects the categories used in the US Renal Data System.

^b^ Other includes American Indian or Alaska Native, Native Hawaiian or Pacific Islander, and multiracial.

Among 2025 patients discharged alive after PE hospitalization, the 1-year recurrence rate was 4.1%. Of 1454 patients (71.8%) with Part D Medicare coverage, only 597 (41%) filled an AC prescription, with similar PE recurrence rates in those who received an AC prescription and those who did not (4.2% vs 2.8%; *P* = .11) (Figure). Inferior vena cava filters were placed in 8% of patients, decreasing from 12.6% in 2011 to 5.1% in 2019 (*P* < .001). Patients who received PD vs HD had a lower 1-year PE incidence (0.61% vs 0.87%, *P* < .001; hazard ratio [HR], 0.68; 95% CI, 0.58-0.80; *P* < .001), but in-hospital and 30-day mortality rates were similar and not statistically significant across modalities.

**Figure.**
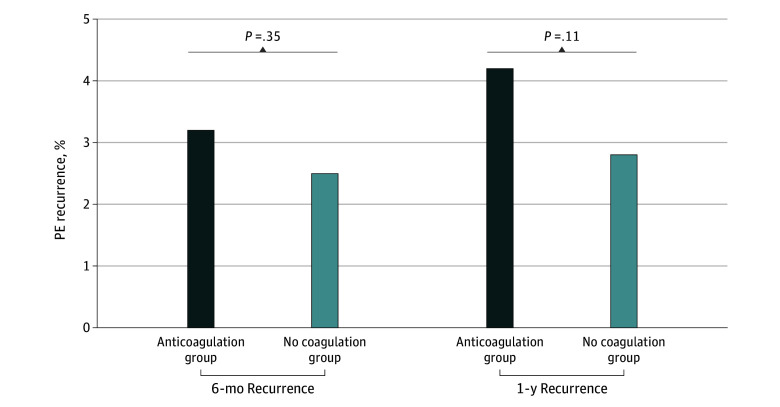
Six-Month and 1-Year Pulmonary Embolism (PE) Recurrence With and Without Anticoagulation Among Patients With End-Stage Kidney Disease Initiating Dialysis

## Discussion

This study highlights the complexity of clinical decision-making regarding AC in patients with ESKD who experience PE. Fewer than half of the patients in this cohort filled postdischarge prescriptions for ACs. Low use of AC in these patients and lack of clear clinical benefit in this study is similar to that seen in patients with ESKD and atrial fibrillation, where the net benefit of AC to prevent stroke or death is uncertain.^3,4^

Patients receiving PD had a significantly lower 1-year PE incidence than those receiving HD even after adjusting for confounders. The mechanism of this finding remains unclear and warrants further investigation. The 1-year incidence of PE in patients with ESKD initiating dialysis from 2011 to 2019 exceeded prior estimates, likely reflecting advancements in imaging for PE detection and possibly improvements in coding practices.^1,5^ The persistently high mortality without significant improvement over time in patients with ESKD compared with the general population reflects the challenges of managing PE in ESKD.^6^

Study limitations include reliance on administrative data, with potential miscoding and lack of clinical granularity. Exclusion of younger patients without Medicare coverage limits generalizability to this subgroup. The low use and unclear benefit of AC following PE underscores the need for further research into the mechanisms of increased thrombotic risk to optimize AC strategies and improve outcomes in this high-risk population.

## Notes

Supplement 1.
eMethods
**eFigure.** Flow chart for patient selection **eTable.** Definitions of study outcomes and adjusted confounders in the multivariable regression model

Supplement 2.
Data Sharing Statement
