# Supplementary material for: Pulmonary Embolism in Patients with End-Stage Kidney Disease Starting Dialysis
Source: JAMA Netw Open. 2025 Mar 17;8(3):e250848. doi: 10.1001/jamanetworkopen.2025.0848 (PMC11915059; doi:10.1001/jamanetworkopen.2025.0848)
Supplement: Supplement 1. — eMethods eFigure. Flow chart for patient selection eTable. Definitions of study outcomes and adjusted confounders in the multivariable regression model [file jamanetwopen-e250848-s001.pdf]

Supplemental Online Content

Patel KN, Chan WC, Bhat V, et al. Pulmonary embolism in patients with end-stage kidney disease starting dialysis. *JAMA Netw Open*. 2025;8(3):e250848. doi: 10.1001/jamanetworkopen.2025.0848

eMethods
eFigure. Flow chart for patient selection
eTable. Definitions of study outcomes and adjusted confounders in the multivariable regression model

This supplemental material has been provided by the authors to give readers additional information about their work.

© 2025 Patel KN et al. *JAMA Network Open*.

eMethods

Data Source:

The study cohort was derived from USRDS files from 2011-2019. The USRDS is a national database that collects, analyzes, and distributes information on all ESKD patients and a selected sample of CKD patients in the US in collaboration with the Centers for Medicare and Medicaid Services (CMS) [1]. The USRDS is funded directly by the National Institute of Diabetes and Digestive and Kidney Disease. The study was reviewed and approved by the University of Kansas Medical Center's Institutional Review Board and USRDS. Informed consent was not required because data were deidentified. We followed Strengthening the Reporting of Observational Studies in Epidemiology (STROBE) reporting guidelines.

Study Design and Selection:

All fee-for-service Medicare beneficiaries with incident ESKD from the USRDS database, between January 1st, 2011, and October 2nd, 2019, were identified. We excluded patients who did not live in the US, patients who were less than 18 years old at the time of starting dialysis, patients who did not have fee-for-service Medicare primary coverage during the first 90 days of starting dialysis, patients who underwent a kidney transplant, and patients who discontinued dialysis within the first 90 days after initiation. Further, we excluded patients with hospitalization records indicating presence of prosthetic heart valves, history of atrial fibrillation/ flutter, prior deep vein thrombosis (DVT)/ PE, or hypercoagulable state during the first 90 days. This was done to exclude patients who may have been on long-term anticoagulation. We also excluded patients with missing information on sex, race, ethnicity, and ESKD cause **(eFigure 1 in Supplement)**. The baseline characteristics were constructed using CMS Form 2728 and hospitalization records, utilizing relevant International Classification of Disease (ICD) codes (9th and 10th versions) within the first 90 days from dialysis initiation. We initiated patient follow-up on day 91 after dialysis initiation, and the follow-up extended until either 1 year from the start of follow-up, censoring, or December 31, 2020, whichever occurred first. Patients were censored if they lost fee-for-service Medicare primary coverage, underwent a kidney transplant, or passed away on or before 1 year from the start of follow-up or by December 31, 2020.

Study Variables:

The demographic variables and comorbidities were identified during the first 90-day enrollment period from the CMS Form 2728 and with the respective ICD diagnosis codes from hospitalizations. Race and ethnicity were derived as reported on CMS form 2728, which is reviewed and signed by both the reporting physician and the patient. Primary outcome was 1-year PE hospitalization incidence after starting dialysis and its yearly trends over the study period. Secondary outcomes were in-hospital mortality, IVC filter placement, 30-day post-discharge mortality, and PE recurrence. 1-year PE hospitalization incidence included patients who were diagnosed with PE and were admitted from the emergency department. 30-day mortality included mortality within 30-days after discharge from the hospital. PE recurrence outcome included readmission during which acute PE was identified. Although some dialysis patients were hospitalized multiple times for pulmonary embolism, only the first instance of PE recorded in the USRDS database was included in the analysis for the PE readmission outcome. 1-year PE hospitalization incidence and PE recurrence were identified by using ICD diagnosis codes of acute PE in the primary diagnosis field. IVC filter placement was identified by using ICD procedure codes in all procedure fields. **eTable 1 in Supplement** lists the outcome definitions.

Statistical Analysis:

After assessing the distribution, continuous variables such as age were presented as the mean and standard deviation due to normal distributions. Categorical variables, such as sex, race, age groups, ESKD cause, dialysis modality, and comorbidities were presented as frequencies with percentages. Continuous variables were compared using a two-sample t-test, and categorical variables were compared using Pearson’s chi-squared test. For post-discharge outcomes, patients who died at the end of index admission were excluded from the analysis. The cumulative incidence rates for PE at 1 year, and PE recurrence were represented using cumulative incidence function (CIF) curves. Death was treated as a competing outcome, and Gray’s test was utilized for comparing HD and PD. To evaluate changes in incidence of PE, IVC filter placement, and clinical outcomes by calendar year, we used logistic regression for categorical variables and linear regression for continuous variables and adjusted with age and sex to find out p-value for trend. We constructed a multivariable hierarchical logistic regression model to compare 1-year PE incidence and in-hospital mortality between HD and PD. We constructed a multivariate Cox regression model to compare 30-day mortality between HD and PD. Multiple patient and hospital covariates were adjusted in the model, and they were selected based on both forward and backward selection models and based on clinical significance to identify predictors for each outcome. We also utilized the Medicare part-D database to assess the anticoagulation prescription fill for the patients who were discharged alive and then

© 2025 Patel KN et al. *JAMA Network Open*.

we created two groups to compare the PE recurrence: anticoagulation (AC) versus no anticoagulation (no AC). The appropriateness of the regression models was assessed by C-statistic, which was >0.85 for all models. All data extraction and analyses used SAS software, version 9.4 (SAS Institute Inc., Cary, NC, USA). Two-sided $p < 0.05$ was used for statistical significance.

References:

1. U.S. Renal Data System. 2022 USRDS annual data report: Epidemiology of kidney disease in the United States. National Institutes of Health, National Institute of Diabetes and Digestive and Kidney Diseases, Bethesda, MD, 2022.

© 2025 Patel KN et al. *JAMA Network Open*.

eFigure. Flow chart for patient selection

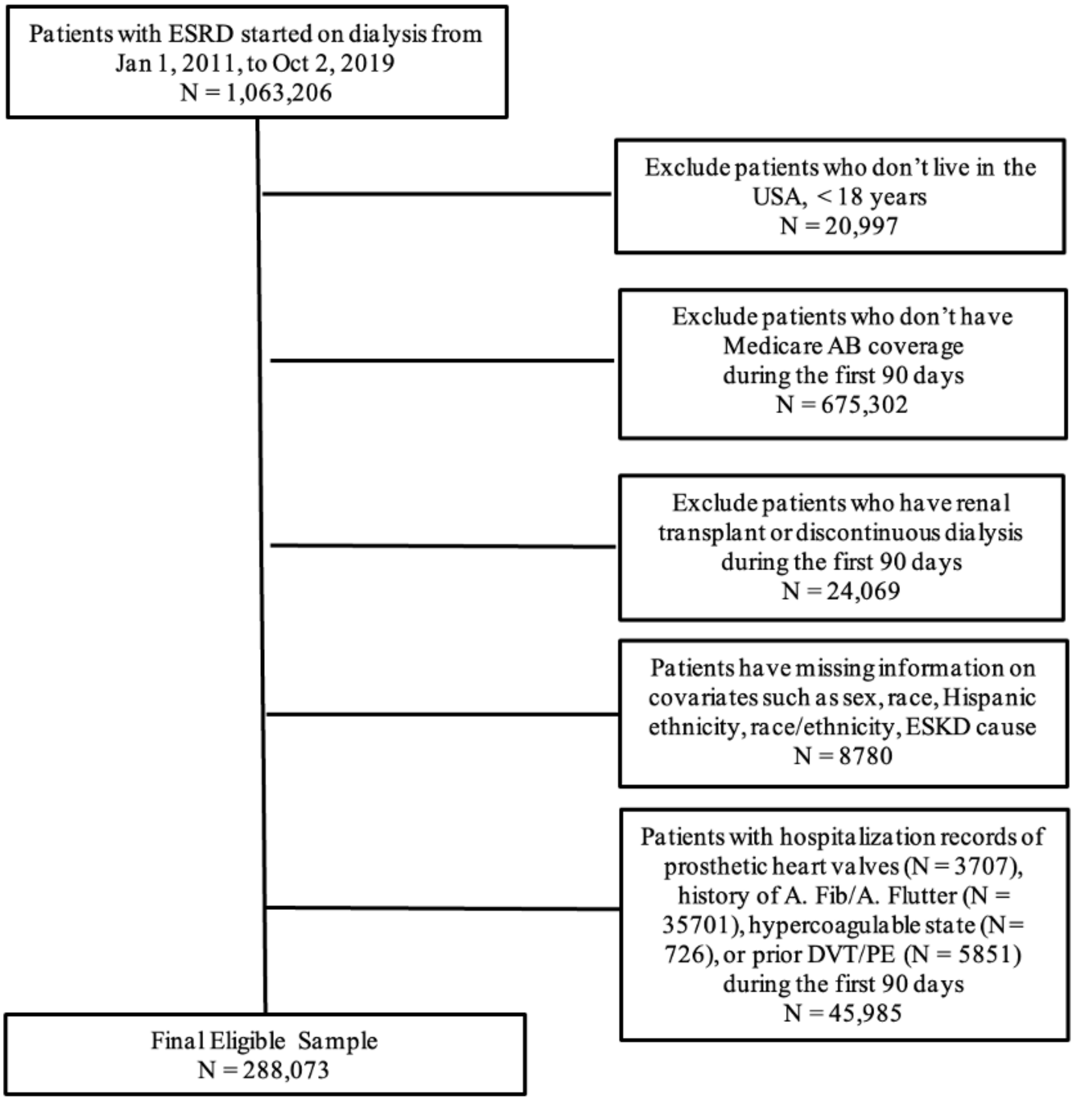

© 2025 Patel KN et al. *JAMA Network Open*.

eTable. Definitions of study outcomes and adjusted confounders in the multivariable regression model

Outcomes	Samples	Regression Methods	Confounders adjusted in the analysis
1-year incidence of PE after starting dialysis in ESKD patients	Overall ESKD patients	Fine-Gray sub distribution hazard model	Sex, race, age group, ESKD cause, all comorbidities
In-hospital mortality in patients with index PE hospitalization	ESKD patients with index PE hospitalization	Logistic regression	Sex, race, age group, ESKD cause, CAD, CHF, PAD, all in-hospital complications
30-day post-discharge mortality in patients with index PE hospitalization	ESKD patients with index PE hospitalization who were discharged alive	Cox regression	Sex, race, age group, ESKD cause, CAD, HF, COPD, cancer, all in-hospital complications

Abbreviations: CAD - coronary artery disease; COPD - chronic obstructive pulmonary disease; ESKD - end-stage kidney disease; HF - heart failure; PE - pulmonary embolism

© 2025 Patel KN et al. *JAMA Network Open*.
