# Supplementary material for: Pulmonary Embolism in Patients with End-Stage Kidney Disease Starting Dialysis
Source: JAMA Netw Open. 2025 Mar 17;8(3):e250848. doi: 10.1001/jamanetworkopen.2025.0848 (PMC11915059; doi:10.1001/jamanetworkopen.2025.0848)
Supplement: Supplement 2. — Data Sharing Statement [file jamanetwopen-e250848-s002.pdf]

Data Sharing Statement

Patel. Pulmonary Embolism in Patients with End-Stage Kidney Disease Starting Dialysis. *JAMA Netw Open*. Published March 17, 2025. doi:10.1001/jamanetworkopen.2025.0848

Data
Data available: No

Additional Information
Explanation for why data not available: The data used in the study were supplied by the Centers for Medicare and Medicaid Services and cannot be rereleased to others. However, investigators could request the data directly from CMS.
